# Stochastic simulations of a synthetic bacteria-yeast ecosystem

**DOI:** 10.1186/1752-0509-6-58

**Published:** 2012-06-06

**Authors:** Konstantinos Biliouris, David Babson, Claudia Schmidt-Dannert, Yiannis N Kaznessis

**Affiliations:** 1 Department of Chemical Engineering and Materials Science, University of Minnesota, 421 Washington Ave SE, Minneapolis, MN 55455, USA; 2 University of Minnesota Biotechnology Institute, 140 Gortner Lab, 1479 Gortner Avenue, Saint Paul, MN 55108, USA; 3 Department of Biochemistry, Molecular Biology and Biophysics, University of Minnesota, 140 Gortner Laboratory, Saint Paul, MN 55108, USA

**Keywords:** Stochastic modeling, Synthetic ecosystem, Cell-cell communication, Synthetic microbial consortia, Bacteria-yeast ecosystem

## Abstract

**Background:**

The field of synthetic biology has greatly evolved and numerous functions can now be implemented by artificially engineered cells carrying the appropriate genetic information. However, in order for the cells to robustly perform complex or multiple tasks, co-operation between them may be necessary. Therefore, various synthetic biological systems whose functionality requires cell-cell communication are being designed. These systems, microbial consortia, are composed of engineered cells and exhibit a wide range of behaviors. These include yeast cells whose growth is dependent on one another, or bacteria that kill or rescue each other, synchronize, behave as predator-prey ecosystems or invade cancer cells.

**Results:**

In this paper, we study a synthetic ecosystem comprising of bacteria and yeast that communicate with and benefit from each other using small diffusible molecules. We explore the behavior of this heterogeneous microbial consortium, composed of *Saccharomyces cerevisiae* and *Escherichia coli* cells, using stochastic modeling. The stochastic model captures the relevant intra-cellular and inter-cellular interactions taking place in and between the eukaryotic and prokaryotic cells. Integration of well-characterized molecular regulatory elements into these two microbes allows for communication through quorum sensing. A gene controlling growth in yeast is induced by bacteria via chemical signals and vice versa. Interesting dynamics that are common in natural ecosystems, such as obligatory and facultative mutualism, extinction, commensalism and predator-prey like dynamics are observed. We investigate and report on the conditions under which the two species can successfully communicate and rescue each other.

**Conclusions:**

This study explores the various behaviors exhibited by the cohabitation of engineered yeast and bacterial cells. The way that the model is built allows for studying the dynamics of any system consisting of two species communicating with one another via chemical signals. Therefore, key information acquired by our model may potentially drive the experimental design of various synthetic heterogeneous ecosystems.

## Background

Advances in the field of synthetic biology have enabled the design of engineered cells performing human-defined functions at a single cell resolution [1,2]. These functions include but are not limited to oscillators [3-5], bistable switches [6], bio-logical gates [7-9], riboregulators [10,11] and molecular devices that control gene expression [12,13]. Despite this progress, several limitations still exist. A major shortcoming is the decreased robustness and the limited potential complexity of single cell functions. Thus, attention has been shifted to synthetic systems based on communication between cells, rather than individual isolated cell functionality. Cooperation among cells is largely mediated by quorum sensing [14] and may be promising for the development of cell-systems that robustly perform complex tasks [15-17]. These tasks range from cells rescuing or killing one another [18-21] to cells synchronizing across a relatively long distance [22].

The potential advantage of microbial consortia compared to monocultures is two-fold. First, in contrast to monocultures, multicultures allow the different species to share the various required synthetic functions or the different steps of a synthetic function. This function sharing decreases the burden in the metabolism of the cells significantly. Second, the sharing of different functions, or steps, among different cells potentially renders microbial consortia more suited for fine-tuning of their artificial functionality [23].

It is now clear that mathematical models can accurately capture the behavior of synthetic systems comprising of either bacterial or yeast cell strains and allowing cell-to-cell communication [18-20,22,24-28]. You and his co-workers designed a synthetic bacterial ecosystem where cell-cell communication controls cell density by inducing a killer gene in the bacteria [19]. To mathematically investigate the dynamics of this system, they coupled their experiments with a simple deterministic model. Shou et al. designed a synthetic yeast system where cell growth was dependent on successful cell-cell communication [24]. To further explain their system behavior, they used a mathematical model comprised of algebraic equations. Basu and his co-workers designed a synthetic system, composed of bacteria, that forms different patterns of differentiation, such as rings and clovers, driven by cell-cell communication via N-Acyl homoserine lactone (AHL) signals [25]. In addition to experimentally designing this system, they used a deterministic mathematical model to explore the behavior of this system. Balagadde et al. designed a synthetic bacterial ecosystem where cell-cell communication enables cells to exhibit predator-prey dynamics by either killing or rescuing one another [18]. They initially developed a deterministic model to thoroughly study the dynamics of their synthetic ecosystem and then introduced a constant noise term to their model aiming to explore the influence of the stochasticity in their system.

Even though communication between different species using non-AHL signals has been demonstrated previously [29], no synthetic ecosystem has been developed that is composed of bacteria and yeast which communicate with and benefit from each other using AHL signals. Such a microbial consortium could exhibit interesting dynamics, such as oscillatory behavior, that stem from the substantial differences (e.g. different volume, growth rate, gene expression process) between prokaryotes and eukaryotes. Here, we investigate the behavior of such a synthetic heterogeneous community using stochastic modeling. To this end, we have modeled and simulated a synthetic consortium composed of *Saccharomyces cerevisiae* (*S. cerevisiae*) and *Escherichia coli* (*E. coli*) cells. This synthetic ecosystem was found to exhibit intriguing dynamic behavior that is commonly observed in natural ecosystems. Our model, capturing the behavior of this ecosystem, has been built in such a way that it can capture the dynamics of any system with two different species communicating with AHL signals. Thus, our model may drive the experimental design of artificial ecosystems with two different species (e.g. mammalian-yeast or mammalian-bacteria) which communicate with and regulate gene expression in one another.

## Methods

### Design of the synthetic ecosystem

In this study, we propose the design of a synthetic yeast-bacteria ecosystem that is based on diffusible chemical signals. Examples of these signals are the RhII/RhlR and LuxI/LuxR quorum sensing signals from *Pseudomonas aeruiginosa* and *Vibrio fisheri* quorum sensing systems, respectively, which are known for their sensitivity and the absence of signal cross-reactivity [30].

**Figure 1:**
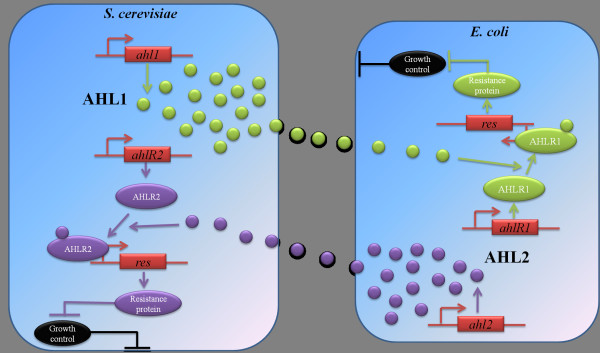
**Logic behind the synthetic yeast-bacteria ecosystem. ***S. cerevisiae* cells produce AHL1 thereby activating resistance gene expression in *E. coli* and cell survival. Similarly, *E. coli* cells produce AHL2 that induces resistance gene expression in *S. cerevisiae* rescuing the latter.

Each species exists in the presence of a molecule controlling growth, Gc. This molecule could be an antibiotic, such as Kanamycin, which is effective against both *E. coli* and *S. cerevisiae*[31]. Gc inhibits cell growth and therefore each species ultimately goes extinct. However, each species contains a resistance gene which counteracts the function of Gc and is controlled by the other species via diffusible molecules. Thus, when both species are present, they induce each other’s resistance gene through chemical signals, thereby rescuing one another. A schematic representation of the proposed ecosystem is illustrated in Figure 1.

More specifically, *S. cerevisiae* constitutively expresses a diffusible molecule, AHL1. AHL1 diffuses out of the *S. cerevisiae* cells, penetrating *E. coli* cells and binding to its cognate receptor, AHLR1. AHLR1 is constitutively produced in *E. coli*. The activated molecule in *E. coli* binds to the responsive element fused upstream of the Res promoter activating expression of Res. Subsequently, the resistance protein, Res, deactivates Gc in *E. coli*. Potential Res could be the Kanamycin resistance protein [31].

The second component of the feedback loop in *E. coli* functions in the same genetic fashion. It constitutively produces an autoinducer synthase, AHL2. Once AHL2 is produced, it diffuses out of the *E. coli* into *S. cerevisiae*, and is recognized by its cognate receptor, AHLR2, which is constitutively produced in *S. cerevisiae* as a fusion protein that allows it to be activated in eukaryotic cells. This activated molecule now binds to its responsive promoter and induces expression of the resistance gene, *res*. The resistance protein, in turn, represses the function of Gc in *S. cerevisiae*.

It is important to note that for the purposes of this study, we assume synthetic bacterial molecular components function in yeast. We hypothesize that their functionality may be retained when they are used in yeast. This is not an unreasonable hypothesis since the functionality of quorum sensing bacterial elements has been demonstrated experimentally in other higher organisms [32].

Here, we aim to computationally explore the behavior of a microbial consortium consisting of two different species, and how the differences of the two species affect its dynamics. The focus is therefore on the population dynamics. The functionality of such an ecosystem could in principle be achieved using any other molecular components with similar function.

### Model description

As discussed in the previous section, numerous mathematical models that describe the behavior of synthetic ecosystems have been developed previously [18-20,24-28]. The vast majority of these models are deterministic, ignoring the stochastic nature which is ubiquitous in biological systems [33-35]. Thus far, different methods have been described [36-42] and extensively applied to stochastically simulate the dynamics of biological systems in general and gene networks in particular [4,8,12,13,43-47].

In this study, we develop a stochastic model that accounts for the intrinsic and extrinsic noise and describes the dynamics of the synthetic bacteria-yeast ecosystem depicted in Figure 1. The model takes into consideration the volume and the growth rate differences between *E. coli* and *S. cerevisiae*. In addition, it accounts for the gene expression dissimilarities between bacteria and yeast. Our model monitors the evolution of molecular species that usually exist in relatively high amounts allowing for the use of continuous stochastic models [38,41]. Continuous stochastic computational approaches have also accurately described the experimental phenotype of synthetic cell communities [18]. We, therefore, employ chemical Langevin equations [41] to capture the evolution of the species participating in this synthetic ecosystem.

The model consists of 17 reactions whose dynamics are described using 9 Stochastic Differential Equations (see Additional file 1). The equations were integrated in Matlab using the Euler Maruyama method [48]. The type of reactions as well as the kinetic parameters used were acquired from previously published studies involving experimental work. Our model is generic (i.e it may be used to capture the dynamics of various two-species ecosystems), but for the purposes of this study we assumed specific molecular components (and their associated kinetic parameters) that have been widely used in designing synthetic ecosystems. These components are presented in Table 1. The current model may capture the behavior of any similar heterogeneous ecosystem by simply modifying the kinetic parameters according to the new system. The reaction network along with the kinetic parameters and the reaction rates capturing the behavior of our system is presented in Table 2.

**Table 1:** Molecular components assumed in the model

**Name**	**Molecular component**
*ahlI*	*rhlI*
*ahlR1*	*rhlR*
AHL1	C4HSL
AHLR1	RhlR
*ahl2*	*luxI*
*ahlR2*	*luxR*
AHL2	3-oxo-C6HSL
AHLR2	LuxR
*res*	*kanR*
Resistance protein (Res)	Kanamycin resistance
Growth control (Gc)	Kanamycin

**Table 2:** Reaction network capturing synthetic ecosystem’s behavior

**#**	**Reaction**	**Reaction rate**	**Kinetic constant**
1	$\overset{k_{1}}{--\to }$*c*_1_	$k_{1}\cdot c_{1}\left(\right)1-\frac{c_{1}+c_{2}}{c_{\text{max}}}))$	$k_{1}=\frac{0.234}{h},c_{\text{max}}=10^{9}\text{cells}$[18,21]
2	$\overset{k_{2}}{--\to }c_{2}$	$k_{2}\cdot c_{2}\left(\right)1-\frac{c_{1}+c_{2}}{c_{\text{max}}}))$	$k_{2}=\frac{0.936}{h}$[19], $c_{\text{max}}=10^{9}\text{cells}$[18,21]
3	$c_{1}+\text{Gc}\overset{k_{3}}{--\to }\text{Gc}$	$\frac{k_{3}^{\prime \prime }\cdot c_{1}}{1+\alpha \cdot \text{Res}1}$	$k_{3}=\frac{4\cdot 10^{6}}{M\cdot h}$[19], $\alpha =\frac{5\cdot 10^{4}}{\text{Molecules}}$
4	$c_{2}+\text{Gc}\overset{k_{3}}{--\to }\text{Gc}$	$\frac{k_{3}^{\prime \prime }\cdot c_{2}}{1+\alpha \cdot \text{Res}2}$	$k_{3}=\frac{4\cdot 10^{6}}{M\cdot h}$[19], $\alpha =\frac{5\cdot 10^{4}}{\text{Molecules}}$
5	$c_{1}\overset{k_{4}}{--\to }\text{AHL}1+c_{1}$	*k*_4_·*c*_1_	$k_{4}=5\cdot 10^{-6}\frac{1}{h}$[18]
6	$c_{2}\overset{k_{5}}{--\to }\text{AHL}2+c_{2}$	*k*_5_·*c*_2_	$k_{5}=5\cdot 10^{-6}\frac{1}{h}$[18]
7	$2\text{AHL}2+2\text{AHLR}2\overset{k_{6}}{--\to }\text{AHL}2:\text{AHLR}2$	$\frac{k_{6}^{\prime \prime }\cdot \text{AHL}2^{2}}{V_{1}\cdot \text{Na}}$	$k_{6}=\frac{3\cdot 10^{19}}{M^{3}\cdot h}$[26], $V_{1}=3.7\cdot 10^{-14}L$[49],
			*Na *= 6.023 · 10^23^
8	$\text{AHL}2:\text{AHLR}2\overset{k_{7}}{--\to }\text{preRes}1$	$\frac{k_{7}\cdot \text{AHL}2:\text{AHLR}2^{n_{1}}}{k_{7b}^{n_{1}}+\text{AHL}2:\text{AHLR}2^{n_{1}}}$	$k_{7}=6\cdot 10^{-5}\frac{M}{h},k_{7b}=10^{-8}M,n_{1}=1$[26]
9	$\text{preRes}1\overset{k_{8}}{--\to }\text{Res}1$	*k*_8_·*preRes*1	$k_{8}=\frac{5}{h}$
10	$2\text{AHL}1+2\text{AHLR}1\overset{k_{9}}{--\to }\text{AHL}1:\text{AHLR}1$	$\frac{k_{9}^{\prime \prime }\cdot \text{AHL}1^{2}}{V_{2}\cdot \text{Na}}$	$k_{9}=\frac{3\cdot 10^{19}}{M^{3}\cdot h}$[26], $V_{2}=10^{-15}L$[50]
11	$\text{AHL}1:\text{AHLR}1\overset{k_{10}}{--\to }\text{Res}2$	$\frac{k_{10}\cdot \text{AHL}1:\text{AHLR}1^{n_{2}}}{k_{10b}^{n_{2}}+\text{AHL}1:\text{AHLR}1^{n_{2}}}$	$k_{10}=6\cdot 10^{-5}\frac{M}{h},k_{10b}=10^{-8}M,n_{2}=1$[26]
12	$\text{AHL}1\overset{k_{11}}{--\to }\emptyset$	*k*_11_·*AHL*1	$k_{11}=\frac{1.19}{h}$[19]
13	$\text{AHL}2\overset{k_{12}}{--\to }\emptyset$	*k*_12_·*AHL*2	$k_{12}=\frac{1.19}{h}$[19]
14	$\text{AHL}1:\text{AHLR}1\overset{k_{13}}{--\to }\emptyset$	*k*_13_·*AHL*1:*AHLR*1	$k_{13}=\frac{1.386}{h}$[26]
15	$\text{AHL}2:\text{AHLR}2\overset{k_{14}}{--\to }\emptyset$	*k*_14_·*AHL*2:*AHLR*2	$k_{14}=\frac{1.386}{h}$[26]
16	$\text{Res}1\overset{k_{15}}{--\to }\emptyset$	*k*_15_·*Res*1	$k_{15}=\frac{4}{h}$[20]
17	$\text{Res}2\overset{k_{16}}{--\to }\emptyset$	*k*_16_·*Res*2	$k_{16}=\frac{4}{h}$[20]

The first two reactions describe the cell population growth. Consistent with previous mathematical models [18-20], and because the model refers to ecology, population growth follows logistic kinetics. Bacteria were considered to grow four times faster than yeast [49]; *k*_1_ was set four times smaller than *k*_2_. *C*_*max *_represents the carrying capacity of the bioreactor, i.e. the maximal population load that the bioreactor can sustain [51], and is set equal to 10^9^ cells [18,21]. Reactions 3 and 4 represent the cell death due to the presence of Gc (in our case Kanamycin). We assume a constant concentration of Gc (0.3 *μM*) as, according to the kinetic parameters used in our model, this concentration kills each single simulated cell colony when the two species are placed separately. Both the bacteria and yeast carry a resistance gene so the reaction rate is written such that the higher the amount of the resistance protein, the slower the cell death rate is. Similar reaction rates have been used previously to capture cell death due to killer proteins [18]. The correlation between Gc and the resistance protein is tuned through the parameter *α*. The parameter *α* was initially set equal to 5 · 10^4 ^molecules^−1^, due to the lack of literature values, and subsequently the sensitivity of the ecosystem’s behavior to changes in this parameter was investigated. Reactions 5 and 6 describe the production of the molecules responsible for the diffusible signals. AHL1 and AHLR2 are produced by *S. cerevisiae* whereas AHL2 and AHLR1 are produced by *E. coli*. The concentration of AHLR2 and AHLR1 is considered constant (0.5 *μM*) and equal to previously published values [26]. AHL1 and AHL2 production reactions are assumed to be first order, in accordance with previous studies [19,20,52]. The production rate of these diffusible molecules can vary significantly depending on the promoter strength of the associated genes. Using directed evolution, a wide range of quorum sensing production rates can be achieved [53]. The optimized behavior can be also achieved using computational approaches [54]. In our model, we initially adopted *k*_4_ and *k*_5_ from [18] and subsequently increased their values since our system required very long time to reach steady state under these conditions. Reaction 7 captures the binding of AHL2 to AHLR2 in *S. cerevisiae*. This reaction is considered a fourth order reaction (this reaction accounts for the volume of *S. cerevisiae* cells) since it has been demonstrated that a fourth order reaction can capture the experimental phenotypes well [26]. Resistance protein (Res) production is calculated using Hill type kinetics, in accordance with experimental observations [26], and is shown in reaction 8. This reaction also accounts for gene expression differences between eukaryotes and prokaryotes. In contrast to prokaryotes, eukaryotic transcription requires many transcription factors to be recruited before its initiation. Moreover, the translation process in prokaryotes is faster than in eukaryotes [55]. These two factors introduce a delay in eukaryotic gene expression rendering it slow compared to the prokaryotic gene expression. In our model, we represent this delay using reaction 8. In fact, we assume that a complex (preRes) must first be formed before Res production can take place. A similar approach has been used previously to capture transcription in yeast [56]. After performing a set of simulations, we set *k*_8_ equal to 5 h^−1^ since this value was found to cause a delay in our ecosystem compared to a model lacking this intermediate reaction (data not shown). The actual process of protein production is captured by reaction 9. Similarly to reaction 7, reaction 10 captures the binding of AHL1 to AHLR1 in *E. coli* (this reaction accounts for the *E. coli* cell volume). Reaction 11 is used to describe *E. coli* gene expression. Note that in this case there is no reaction describing a delay in gene expression. Finally, reactions 12-17 represent the degradation of the species participating in this network and they are all considered first-order.

## Results and discussion

### Testing synthetic ecosystem’s functionality

Initially, we explored whether *S. cerevisiae* cells can withstand Gc in the absence of *E. coli* cells and vice versa. We simulated the behavior of 50,000 yeast cells and 50,000 bacterial cells in the absence of Gc. Our simulations indicated that both *S. cerevisiae* and *E. coli* grow normally (data not shown). However, when each of the two populations is placed in a simulated bioreactor separately, in the presence of Gc, neither population is able to survive (data not shown).

**Figure 2:**
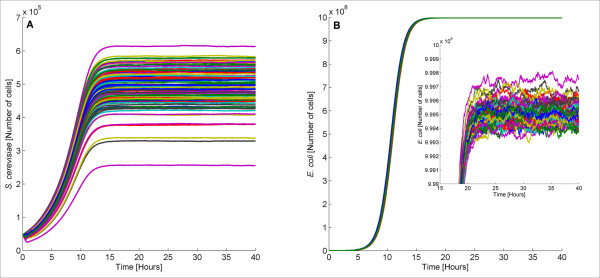
**Behavior of coexisting *S. cerevisiae *and *E. coli *cells.** When the two species are placed together, obligatory mutualism is observed, i.e they benefit from each other and survive from Gc. The inset represents part of Figure 2**B** and shows the fluctuations of *E. coli* population size.

Subsequently, we simulated the behavior of bacteria and yeast when both are present to test whether communication and cooperation between these two species can be successfully achieved. We simulated stochastically 100 different population colonies containing 50,000 *E. coli* and 50,000 *S. cerevisiae* cells. The results are depicted in Figures 2A (yeast cells) and 2B (bacterial cells). The different lines correspond to the cell population size in different trajectories. The evolution of all the species is provided in the Additional file 1.

In both cases, the two different species exploit communication with one another for successful survival in the presence of Gc. Our simulations demonstrate that yeast can successfully induce the expression of the resistance gene found in bacteria and vice versa. This is a common characteristic of ecosystems called obligatory mutualism. In other words, *S. cerevisiae* and *E. coli* cells are not able to survive separately but they are able to grow in concert. As expected, we observe that the number of *E. coli* cells is always higher than the number of *S. cerevisiae* cells. As discussed before, the reason for this is the high growth rate of bacteria relative to yeast.

Variation regarding the number of cells is observed when different colonies are simulated and is attributed to the stochasticity underlying biological functions. The average *S. cerevisiae* population (calculated over 100 trajectories) is around 4.90· 10^5^ cells and the standard deviation is equal to 5.77· 10^4^ cells. The mean *E. coli* population is about 9.99·10^8^ cells and the standard deviation is approximately 6.68·10^4^ cells. Note that the total number of cells cannot exceed 10^9^. Both bacteria and yeast require approximately 16 hours to reach steady state. In every single bioreactor, neither bacteria nor yeast die from the presence of Gc. This demonstrates that communication can take place between *S. cerevisiae* and *E. coli* allowing for the survival of the two species.

Even though Figure 2 establishes cell communication and obligatory mutualism between *E. coli* and *S. cerevisiae* cells, this refers only to the case described by this set of parameters. Thus, in order to investigate which parameters promote successful communication and cooperation between *E. coli* and *S. cerevisiae* cells, and to explore the dynamics of different parameter sets, a sensitivity analysis was performed. To implement this, we systematically modified different parameters within reasonable ranges and monitored the dynamics of the system. In what follows, we present the evolution of the average *S. cerevisiae* and *E. coli* population over 100 trajectories. In some cases, we further provide all the 100 trajectories with variation in the values of key parameters examined in our analysis.

### Ecosystem’s sensitivity to parameter *α*

**Figure 3:**
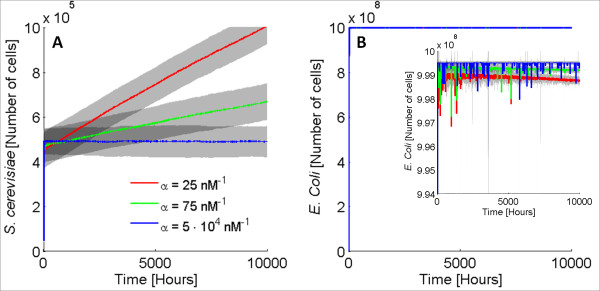
**Average values and standard deviation of *S. cerevisiae *(A) and *E. coli *(B) population for different values of *α.*** Mean values and standard deviation (grey shade) of 100 trajectories of *S. cerevisiae* (**A**) and *E. coli* (**B**) population size for different values of the parameter *α*.

As discussed in the previous section, *α*represents a correlation between the molecule controlling growth and the resistance protein. More specifically, the larger the *α*, the smaller the amount of Res required for cells to survive from Gc (see Table 2). Since this parameter is of high importance for our model, and because it was the only parameter not acquired from previously published models, we explored the influence of *α* on the system’s behavior. To this end, we performed multiple computational experiments modifying *α*and investigating our ecosystem’s dynamic behavior. Our simulation results showed that when *α* is larger than 100 nM^−1^, the total system’s behavior does not change appreciably (data not shown). For values of *α* smaller than 0.07 nM^−1^, the ecosystem is driven to extinction (data not shown). Importantly, our simulations’ data demonstrated that for values of *α* in the range of 0.07 nM^−1^ to 100 nM^−1^, the dynamics of the system, and specifically the time the system needs to reach steady state, becomes remarkably slow. Figures 3A and 3B show the mean values, along with the standard deviation, of 100 trajectories from the stochastic simulations for *α* equal to 25 nM^−1^ (red), 75 nM^−1^(green) and 5· 10^4^ nM^−1^(blue). As observed in Figure 3, when the value of *α*is lower than 100, the system cannot reach steady state even after 10,000 hours. To our knowledge, no synthetic ecosystem exists that reaches steady state after such a long time suggesting that in order for this system to be realistic, the value of *α* in our model should be higher than 100 nM^−1^. This is confirmed by the fact that our system reaches steady state approximately as fast as previously published synthetic ecosystems systems did [18-20].

### Importance of Gc concentration

**Figure 4:**
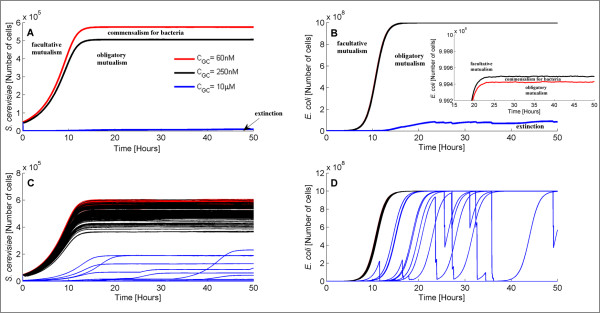
**Average values and single trajectories of *S. cerevisiae *and*E. coli *population for different Gc concentrations.** Average (over 100 trajectories) values (**A,B**) and 100 single trajectories (**C,D**) of *S. cerevisiae* and *E. coli* population size for Gc concentration equal to 60 nM (red), 250 nM (black) and 10 *μM*(blue). The synthetic ecosystem adopts different behaviors, that are commonly observed in natural ecosystems, in response to different Gc concentrations.

Previous studies describing similar synthetic ecosystems have demonstrated the importance of the concentration of the molecule controlling growth on the system’s dynamics [20]. Guided by this, we conducted a set of simulations where we modified Gc’s concentration. We monitored the dynamics of the system for three different Gc concentrations. The average population values (over 100 trajectories) for each concentration are shown in Figures 4A and 4B. 100 trajectories of the two species population for the different Gc concentrations are provided in Figures 4C and 4D.

As Figure 4 indicates, an increase on Gc’s concentration from 60 nM to 250 nM is followed by a decreased yeast population and an increased bacterial population. In other words, upon increasing Gc concentration in the bioreactor, *E. coli* cells benefit whereas *S. cerevisiae* cells are harmed. Based on the way our model was built, this is likely ascribed to the fact that yeast grow much slower than bacteria and can therefore resist only low Gc concentrations. As the antibiotic concentration increases, yeast die faster than bacteria and the latter, even though they grow slower than they would in the absence of Gc, take advantage of the higher nutrient levels in the bioreactor. This is an interesting characteristic and could be used as a means for controlling the bioreactor’s population, obviating the need of adding or removing cells. However, when Gc concentration is significantly high (e.g. 10 *μ*M), the average value (of the 100 trajectories) of both populations decreases dramatically as many single trajectories reach zero.

Further analysis of the system’s behavior indicated that changing the Gc concentration leads to an intriguing behavior commonly exhibited by natural ecosystems. More specifically, when Gc levels are low, each species can survive even in the absence of the other species. In particular, bacterial cells can withstand up to 250 nM Gc. On the other hand, yeast cells cannot survive even these Gc levels and they can only withstand Gc concentrations lower than 60 nM. Having said this, the behavior of the system for Gc levels up to 60 nM is analogous to facultative mutualism, i.e. both species benefit from but are not dependent on each other. However, when Gc’s level lies between 60 nM and 250 nM, the behavior of the system is similar to commensalism for bacteria, i.e. bacteria can survive without yeast but yeast are not able to survive without bacteria.

The lethal Gc concentration for cultures with both cell types present is 20 *μ*M. Based on this, we conclude that when Gc’s levels are between 250 nM and 20 *μ*M, the behavior of the system is homologous to obligatory mutualism as both species are completely dependent on each other and unable to survive individually. Finally, for Gc levels higher than 20 *μ*M, we observe ecosystem’s extinction. Such behaviors have been observed previously in similar synthetic bacterial ecosystems [20] and are shown in Figure 4. The concentrations used in Figure 4 represent the boundaries between different system’s behavior (note that instead of 20 *μ*M Gc, which is the boundary between obligatory mutualism and extinction, we considered 10 *μ*M Gc). The population dynamic behavior for Gc concentrations between the ones used here lies in the area between these lines.

Figures 4C and 4D demonstrate deviation among the different cell density trajectories. Note that this deviation could not be captured using deterministic simulations. The standard deviation (calculated over the 100 trajectories) of the population size at 50 hours and for 60 nM, 250 nM, and 20 *μ*M Gc is shown in Table 3.

**Table 3:** Standard deviation of population size at steady state for different Gc concentrations

**Gc [nM]**	***S. cerevisiae *[10**^**4**^**cells]**	***E. coli *[10**^**4**^**cells]**
60	1.42	3.55
250	4.93	5.61
2·10^4^	3.87	2.77

### Ecosystem’s sensitivity to various carrying capacities and initial cell densities

**Figure 5:**
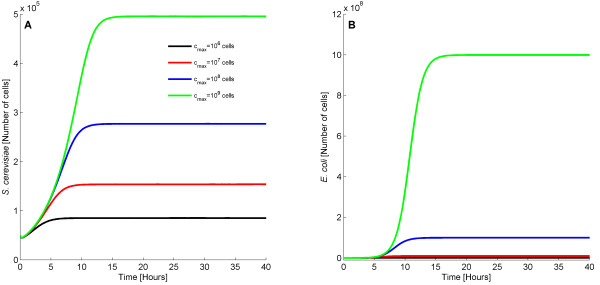
**Average (over 100 trajectories) *S. cerevisiae *(A) and *E. coli *(B) population size for different *c***_***max***_**.** Average (over 100 trajectories) values of *S. cerevisiae* (**A**) and *E. coli* (**B**) population size for different reactor capacities. The higher the reactor capacity the higher the steady state population density of the two species is.

We then explored the influence of *c*_*max *_and the initial cell population on the synthetic ecosystem’s behavior. As discussed above, the carrying capacity is the maximum number of (bacterial and yeast) cells that can exist in the bioreactor [51]. Here, we only show average values of the 100 trials since the single trajectories exhibit similar behavior as in the previous cases. Figures 5A (*S. cerevisiae*) and 5B (*E. coli*) show average population sizes for different *c*_*max *_values.

As expected, an increase in *c*_*max *_causes an increase on both yeast and bacterial steady state populations as the nutrients in the culture suffice for more cells. Thus, both species grow faster and consequently survive in the presence of Gc. It is important to note that a minimum amount of nutrients must exist in the bioreactor for the cells to grow and survive. Thus, we ran simulations decreasing *c*_*max*_ to find this minimum threshold under which the ecosystem goes extinct. According to our simulation results, the minimum *c*_*max*_ in order for all the trajectories to end up in non-zero steady states (over a period of 3,000 hours) is equal to approximately 2 · 10^5^ cells (data not shown). Thus, the model suggests that our synthetic ecosystem is fully functional only for reactor capacities equal to or higher than 2 · 10^5 ^cells.

As in the previous cases, deviation among the different population trajectories was observed. The steady state standard deviation of the population size for various reactor capacities is provided in Table 4. Notably, the larger the reactor capacity, the higher is the deviation among the different population trajectories.

**Table 4:** Standard deviation of population size at steady state for different reactor capacities

***C***_***max***_**[cells]**	***S. cerevisiae *[10**^**4**^**cells]**	***E. coli *[10**^**4**^**cells]**
10^6^	1.14	1.14
10^7^	1.96	1.91
10^8^	3.20	3.10
10^9^	5.73	6.60

We further explored the minimum initial number of total cells required in order for the two species population to cooperate favorably and survive. To do so, we performed different simulations starting with equal *E. coli* and *S. cerevisiae* populations and monitoring the system’s dynamics for 1,000 hours. Our results showed that for equal initial populations of the two species, the minimum number of *S. cerevisiae* and *E. coli* cells in the reactor should be approximately equal to 15 cells for the ecosystem to survive with Gc. Moreover, when the initial *E. coli* population is 50,000 cells, the minimum *S. cerevisiae* initial population required in order for the system to avoid extinction is 14 cells. On the other hand, when the initial *S. cerevisiae* population is 50,000 cells, the required *E. coli* initial population is 4 cells. This difference is ascribed to the fact that bacteria grow predominantly fast thereby quickly helping yeast to survive and therefore only 4 yeast cells are initially required to make the ecosystem functional. However, yeast grow and consequently rescue *E. coli* with a slower rate and therefore larger *E. coli* population is initially required for the ecosystem to function.

### Effects of *E.coli* cell death rates on the ecosystem’s dynamics

It is clear from the aforementioned analysis that in most cases bacterial cell populations dominate yeast cell populations because of their high growth rate. We therefore introduced a bacteria degradation term in our network to enhance the competition between the population of the two species. We only considered *E. coli* degradation as bacteria grow significantly faster than yeast. This degradation could be achieved experimentally as bacteria can be engineered to stimulate their lysis in response to a human-defined signal. More specifically, introducing holin and lysozome genes that are activated via AHL signals, allows for controlling cell membrane destruction and consequently cell death [57].

Initially, we performed our analysis under the assumption that the deterministic term dominates the stochastic term, i.e. the intrinsic noise of the system is negligible. The results presented in what follows were therefore produced based only on the deterministic part of the equations 1-9. A similar approach has been used previously to explore the oscillatory behavior of a synthetic ecosystem [18].

As expected, high degradation rates cause bacterial cell death followed by yeast wash out due to obligatory mutualism (data not shown). In contrast, low degradation rates allow yeast domination, as bacterial populations quickly decreases due to both Gc and degradation, thereby allowing an increase in yeast population (data not shown).

**Figure 6:**
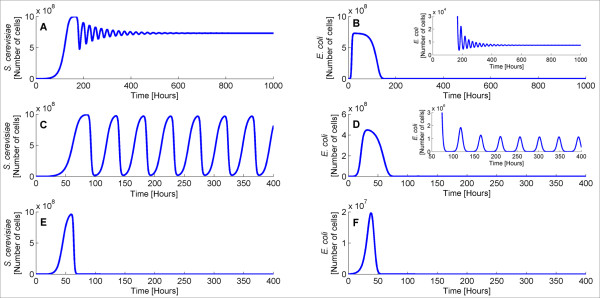
***S. cerevisiae *(A,C,E) and *E. coli *(B,D,F) population dynamics for different *E. coli *degradation rates. ***S. cerevisiae* (**A,C,E**) and *E. coli* (**B,D,F**) population dynamics for *E. coli* degradation rate equal to 0.25, 0.50 and 0.75 h^−1^. For d = 0.25 h^−1^(**A,B**) the ecosystem exhibits damped oscillations. For d = 0.50 h^−1^(**C,D**) the population of the two species oscillates with sustained oscillations whereas for d = 0.75 h^−1^(**E,F**) goes to zero.

Importantly, and as observed in other synthetic ecosystems composed of species with different growth rates [18], there is a range of bacterial degradation rate where *S. cerevisiae* and *E. coli* population exhibit sustained oscillations. These oscillations originate from the antagonism between the two species population and demonstrate a predator-prey like relationship between *S. cerevisiae* and *E. coli* cells. In particular, when the *E. coli* degradation rate, d, lies between 0.30 h^−1^ and 0.72 h^−1^, sustained oscillations are observed. For d smaller than 0.30 h^−1^, damped oscillations are exhibited. Finally, for d larger than 0.72 h^−1^, the two species population goes to zero. In other words, the ecosystem becomes extinct, since this high degradation rate results in bacterial death which in turn leads to yeast extinction because cell-cell communication cannot take place favorably anymore. Figure 6 shows the behavior of the two species population for degradation parameters that lie in the aforementioned ranges. When d is equal to 0.25 h^−1^(Figures 6A, 6B), we observe damped oscillations that end up on a stable steady state. However, from d = 0.30 h^−1^ to d = 0.72 h^−1^, sustained oscillations whose amplitude scales with the degradation rate are observed. This trend is provided in Figures 6C, 6D where d = 0.50 h^−1^. Finally, when d is larger than 0.72 h^−1 ^the system is driven to extinction, as depicted in Figures 6E and 6F.

**Figure 7:**
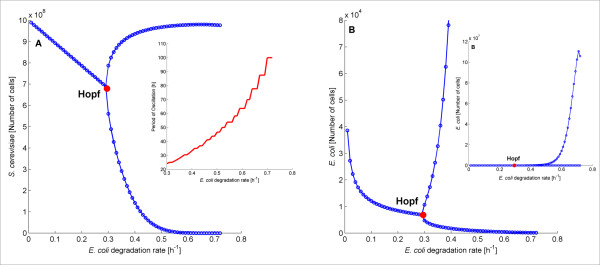
**Bifurcation diagram of the *S. cerevisiae *(A) and *E. coli *(B) population versus the *E. coli *degradation rate.** Bifurcation diagram of the *S. cerevisiae* (**A**) and *E. coli* (**B**) population versus the degradation rate of *E. coli* cells. For the sake of clarity, Figure B shows only part of the bifurcation diagram whereas the complete bifurcation diagram is illustrated in the inset. The period of oscillation of *S. cerevisiae* and *E. coli* cells for different *E. coli* degradation rates is the same and presented as inset in Figure 7A.

The bifurcation diagram describing our ecosystem’s oscillatory behavior is presented in Figure 7. A Hopf point, where sustained oscillations of the two species population initiate, is observed for d approximately equal to 0.30 h^−1^(red). The Hopf point was further confirmed by eigenvalue analysis. The lines following the Hopf point correspond to the oscillation amplitude, as calculated from the transient analysis. Please note that for the sake of clarity, in Figure 7B we show the upper limit of oscillations only for d up to 0.41 h^−1^. The complete bifurcation diagram is provided in the inset. The period of the oscillations was calculated using the FFT (Fast Fourier Transform) function in Matlab and is depicted as inset in Figure 7A. As evident, the period of the oscillations scales with the *E. coli* degradation rate. This is an intriguing observation which suggests that the *E. coli* degradation rate could be used to control the period of our oscillatory ecosystem.

**Figure 8:**
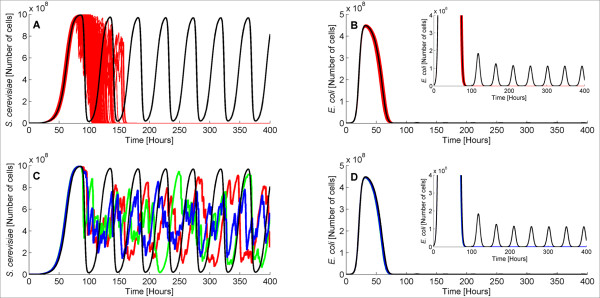
**Population density of *S. cerevisiae *and *E. coli*for d = 0.5 h*−***^**1**^**calculated using stochastic and deterministic simulations.** A,B: Population size of *S. cerevisiae* (**A**) and *E. coli* (**B**) for d = 0.50 h^−1^calculated using stochastic (red) and deterministic simulations (black). C,D: Population size of *S. cerevisiae* (**C**) and *E. coli* (**D**) for d = 0.50 h^−1^calculated with deterministic (black) and stochastic (red, green, blue) simulations with 1.25% of the current intrinsic noise terms.

It should be stressed that including the stochastic terms in our simulations, leads to the ecosystem’s extinction. This has been observed before [18] and is caused by the fact that during the oscillations, the bacterial population reaches small values and therefore noise terms destroy the sustained oscillations by driving bacterial population to zero and consequently the ecosystem to extinction (since cooperation cannot occur). In fact, the smaller the noise amplitude, the higher the probability for the system to circumvent extinction and exhibit sustained oscillations. Figure 8 shows a comparison between deterministic and stochastic simulations. For d = 0.50 h^−1^(Figures 8A and 8B), deterministic solution (black) provides sustained oscillations that end up in a steady state. Stochastic simulations (red) are consistent with the deterministic ones, i.e. demonstrate oscillations, but only for a small period of time and subsequently all the trajectories reach zero. Motivated by this observation, we performed several simulations (for d = 0.50 h^−1^) where we systematically decreased the noise terms amplitude. Our simulations demonstrated that when the noise terms are 1.25% or less of the current values, the stochastic behavior matches the deterministic one, i.e. the ecosystem population exhibits sustained oscillations. When the noise terms are between 1.30% and 100% of the current values, there are always stochastic trajectories that reach zero over a period of 1,000 hours. Figures 8B and 8C show three population density trajectories of the stochastic simulation (green, red, blue) compared with the deterministic simulation (black) when the noise terms are reduced to 1.25%. As evident, the ecosystem’s behavior provided by the two approaches is consistent and stochastic trajectories exhibit continuous oscillations.

Overall, our simulations suggest that high amplitude intrinsic noise damages the ecosystem’s oscillatory behavior. On the other hand, less noisy environments stimulate the sustained oscillation of the two species population.

## Conclusions

We presented the *in silico* design of the first synthetic bacterial-yeast ecosystem where communication between cells is achieved using AHL signals. The model, while developed to accurately depict these interactions, can be adapted to characterize any cell-to-cell communication and population dynamics mediated by diffusible chemical signaling.

We showed that when the two species coexist, they overcome Gc’s toxicity by inducing each other’s resistance gene via small molecule signalling and therefore survive. Our simulations suggest that the minimum reactor capacity required for this ecosystem to evolve is 2 · 10^5^cells. By varying the Gc concentration, the ecosystem adopts different behaviors including obligatory and facultative mutualism, commensalism and extinction. Adding an *E. coli* degradation reaction, which can be experimentally realized by engineering bacteria to induce lysis, can drive the population of the two species to predator-prey like dynamics, i.e. sustained oscillations. These oscillations can, however, be destroyed in noisy environments. Overall, we demonstrated that such kind of heterogeneous synthetic ecosystems could exhibit interesting dynamics.

As demonstrated here and in different studies [18], the development of synthetic microbial consortia using species with different characteristics (e.g. different growth rate or volume) yields systems with intriguing dynamics, such as oscillations. These systems could have various potential applications such as the delivery of two different drugs in dissimilar time intervals [23].

Our mathematical model may potentially drive the experimental design of microbial consortia with a heterogeneous population. This and similar mathematical models can further be used to predict interspecies bioreactor dynamics under numerous conditions, with differing chemical signals, and employing various population control mechanisms. Engineered interspecies system have substantial implications for complex chemical synthesis as well as future biorefinery design and optimization. Thus, the dynamics analysis presented herein may be used as the basis for the *in vivo* design of such promising synthetic ecosystems.

## Competing interests

The authors declare that they have no competing interests.

## Authors contributions

KB developed the model, carried out the simulations and wrote the manuscript. DB participated in the design of the study. CSD conceived of the study and participated in its design. YK conceived of the study, participated in its design and coordination and helped to write the manuscript. All authors read and approved the final manuscript.

## Supplementary Material

Additional file 1 **Stochastic differential equations used to simulate the behavior of the synthetic ecosystem.** This file contains the equations and the species used to stochastically simulate the behavior of the synthetic ecosystem. It also includes 100 trajectories of the evolution of all the species when *S. cerevisiae* and *E. coli* coexist (In support of Figure 2). Click here for file
