# Supplementary material for: Stochastic simulations of a synthetic bacteria-yeast ecosystem
Source: BMC Syst Biol. 2012 Jun 6;6:58. doi: 10.1186/1752-0509-6-58 (PMC3485176; doi:10.1186/1752-0509-6-58)
Supplement: Additional file 1 — Stochastic differential equations used to simulate the behavior of the synthetic ecosystem. This file contains the equations and the species used to stochastically simulate the behavior of the synthetic ecosystem. It also includes 100 trajectories of the evolution of all the species when S. cerevisiae and E. coli coexist (In support of Figure 2). [file 1752-0509-6-58-S1.pdf]

Additional File 1

March 5, 2012

Species

$y_1 = S.\ cerevisiae\ cells$
$y_2 = E.\ coli\ cells$
$y_3 = C4HSL$
$y_4 = 3oxoC6HSL$
$y_5 = preKanR1$
$y_6 = KanR1$
$y_7 = 3oxoC6HSL : LuxR$
$y_8 = C4HSL : RhlR$
$y_9 = KanR$

Stochastic Differential Equations capturing the reaction network dynamics

$$dy_1 = \left(k_1 y_1 \left(1 - \frac{y_1 + y_2}{c_{max}}\right) - \frac{k_3 \cdot Kan \cdot y_1}{1 + \alpha \cdot y_6}\right) dt + \sqrt{k_1 y_1} dw_1 - \sqrt{k_1 y_1 \frac{y_1 + y_2}{c_{max}}} dw_2 - \sqrt{\frac{k_3 \cdot Kan \cdot y_1}{1 + \alpha \cdot y_6}} dw_3$$

$$dy_2 = \left(k_2 y_2 \left(1 - \frac{y_1 + y_2}{c_{max}}\right) - \frac{k_3 \cdot Kan \cdot y_2}{1 + \alpha \cdot y_9}\right) dt + \sqrt{k_2 y_2} dw_4 - \sqrt{k_2 y_2 \frac{y_1 + y_2}{c_{max}}} dw_5 - \sqrt{\frac{k_3 \cdot Kan \cdot y_2}{1 + \alpha \cdot y_9}} dw_6$$

$$dy_3 = \left(k_4 y_1 - \frac{k_9 \cdot RhlR^2 \cdot {y_3}^2}{Na \cdot V_2} - k_{11} y_3\right) dt + \sqrt{k_4 y_1} dw_7 - \sqrt{\frac{k_9 \cdot RhlR^2 \cdot {y_3}^2}{Na \cdot V_2}} dw_8 - \sqrt{k_{11} y_3} dw_9$$

$$dy_4 = \left(k_5 y_2 - \frac{k_6 \cdot LuxR^2 \cdot {y_4}^2}{Na \cdot V_1} - k_{12} y_4\right) dt + \sqrt{k_5 y_2} dw_{10} - \sqrt{\frac{k_6 \cdot LuxR^2 \cdot {y_4}^2}{Na \cdot V_1}} dw_{11} - \sqrt{k_{12} y_4} dw_{12}$$

$$dy_5 = \left(\frac{k_7 y_7^{n_1}}{k_{7b}^{n_1} + y_7^{n_1}} - k_8 y_5\right) dt + \sqrt{\frac{k_7 y_7^{n_1}}{k_{7b}^{n_1} + y_7^{n_1}}} dw_{13} - \sqrt{k_8 y_5} dw_{14}$$

$$dy_6 = \left(k_8 y_5 - k_{15} y_6\right) dt + \sqrt{k_8 y_5} dw_{14} - \sqrt{k_{15} y_6} dw_{15}$$

$$dy_7 = \left(\frac{k_6 \cdot LuxR^2 \cdot {y_4}^2}{Na \cdot V_1} - k_{14} y_7\right) dt + \sqrt{\frac{k_6 \cdot LuxR^2 \cdot {y_4}^2}{Na \cdot V_1}} dw_{11} - \sqrt{k_{14} y_7} dw_{16}$$

$$dy_8 = \left(\frac{k_9 \cdot RhlR^2 \cdot {y_3}^2}{Na \cdot V_2} - k_{13} y_8 \right) dt + \sqrt{\frac{k_9 \cdot RhlR^2 \cdot {y_3}^2}{Na \cdot V_2}} dw_8 - \sqrt{k_{13} y_8} dw_{17}$$

$$dy_9 = \left(\frac{k_{10} y_8^{n_2}}{k_{10b}^{n_2} + y_8^{n_2}} - k_{16} y_9 \right) dt + \sqrt{\frac{k_{10} y_8^{n_2}}{k_{10b}^{n_2} + y_8^{n_2}}} dw_{18} - \sqrt{k_{16} y_9} dw_{19}$$

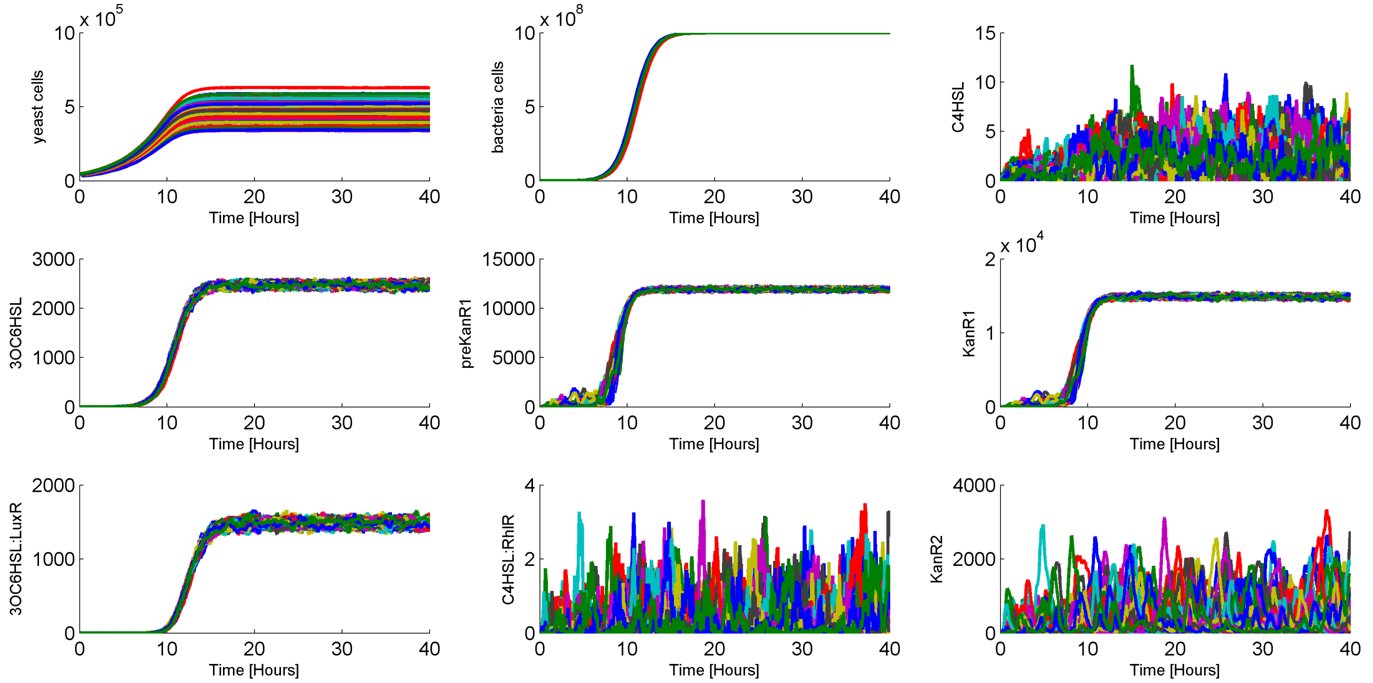

Figure 1: 100 trajectories of the evolution of all the species when yeast and bacteria coexist (In support of Figure 2).
